# AI-driven transformation in forensic medicine education: applications, pedagogical shifts, and future challenges

**DOI:** 10.3389/fmed.2026.1732967

**Published:** 2026-01-29

**Authors:** Yin-qi Wu, Yu Du

**Affiliations:** School of Forensic Science and Technology, Criminal Investigation Police University of China, Shenyang, China

**Keywords:** artificial intelligence, forensic medicine education, personalized learning, teaching reform, virtual simulation

## Abstract

Forensic medicine, as an interdisciplinary field featuring a high degree of practicality and technological integration, is currently faced with several challenges, including limited teaching resources, few practical training opportunities, and slow adoption of emerging technologies. With the rapid advancement of artificial intelligence (AI), its growing application in medical education has significantly transformed the landscape of traditional teaching approaches in forensic medicine, opening up new possibilities for innovations in pedagogical models. Against this backdrop, this study examines the potential of AI technology in forensic medicine from the perspectives of pedagogical model innovation, content delivery, and evaluation system reform, with specific emphasis on virtual simulation instruction, intelligent case analysis, and personalized learning pathways. Furthermore, it discusses existing ethical, technical, and resource-related limitations in current research. Based on this study, the paper proposes strategies and practical pathways to promote the application of AI in forensic medicine education, aiming to provide theoretical insights for cultivating high-quality forensic medicine professionals.

## 1 Introduction

In 1946, the advent of the first computer marked the emergence of AI, which began to replace humans in performing certain cognitive tasks (1). However, due to the limited technological capabilities of these early systems, AI’s early progress was slow. It was not until the 1990s that AI entered a phase of rapid innovation, driven by the rapid advancement of Internet technologies and resulting in systems that began to surpass human performance in specific domains (2). In recent years, breakthroughs in information technologies such as big data and cloud computing have ushered AI into a period of dramatic growth. Generative AI models, including ChatGPT, DeepSeek, and ERNIE Bot, have been widely adopted across industries such as academic research, healthcare, and education, significantly enhancing efficiency while reducing labor costs. Moreover, AI has found extensive applications in medical education, demonstrating highly effective outcomes in areas such as teaching assistance, management, and simulation-based practical training.

This potential for transformation is especially pertinent to forensic medicine. As an interdisciplinary field integrating medicine, law, and physical evidence technology-with sub-disciplines such as forensic physical evidence, forensic pathology, crime scene investigation, forensic anthropology-forensic medicine education faces distinct and persistent challenges. The traditional teaching model has long been constrained by the scarcity of realistic practical scenarios, reliance on outdated technical tools that hinder engagement and teaching effectiveness, and a rigid evaluation system overly focused on attendance and exam grades, thereby neglecting dynamic assessments of students’ comprehensive anlytical abilities and practical qualities (3–5). These limitations make forensic medicine a prime candidate for AI-driven innovation. The integration of artificial intelligence holds the potential to address these limitations and attain pedagogical outcomes unattainable using traditional methods. Based on this, this article will explore the reform of forensic medicine teaching in terms of pedagogical models, content delivery, and evaluation systems focusing on applications such as virtual simulation experiments on teaching, intelligent case-based learning, personalized learning pathways, etc., while analyzing their development prospects and existing problems.

This paper will comprehensively review the application of AI technology in forensic medical education to examine how AI can systematically address the longstanding limitations of traditional teaching in forensic medicine education-specifically in enhancing practical training, modernizing content delivery, and transforming assessment methods—and to propose actionable pathways for its integration. Hopefully, this review can provide a detailed and comprehensive reference for the future development of forensic science education and promote the integration of forensic medicine education with artificial intelligence technology.

## 2 Review methodology

**FIGURE 1:**
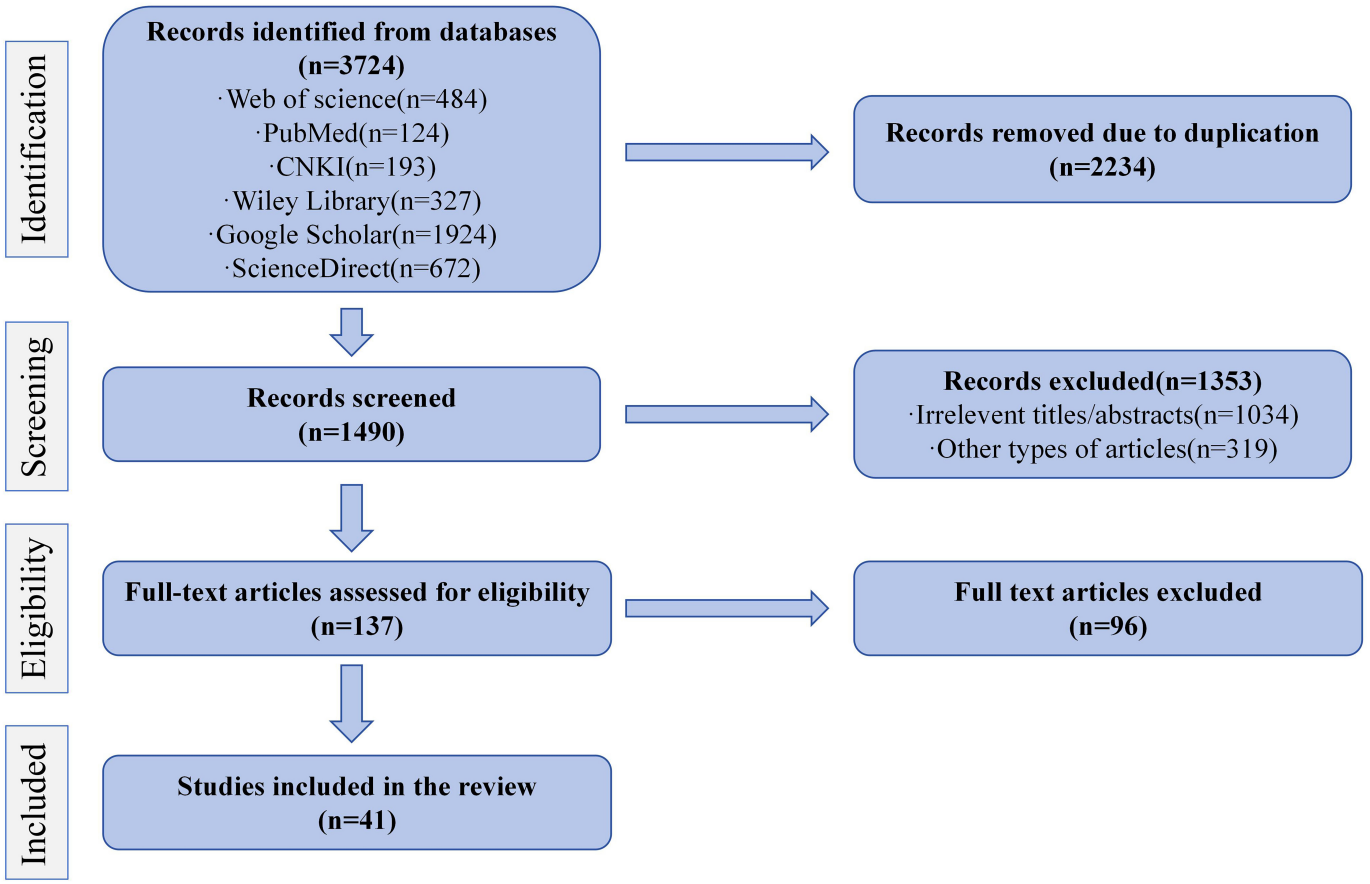
The systematic literature search process, including identification, screening, eligibility, and inclusion.

We conducted an extensive literature search across major electronic databases such as PubMed,^1^ CNKI,^2^ Google Scholar,^3^ Science Direct,^4^ Wiley Online Library,^5^, and Web of Science,^6^ using keywords such as forensic science, forensic anthropology, forensic pathology, forensic scene investigation, forensic physical evidence, education, virtual reality, 3D imaging, artificial intelligence, and so on. After removing 2,234 duplicate records, we screened 1,490 articles based on their titles and abstracts. This initial screening phase led to the exclusion of 1,353 records, comprising 1,034 studies deemed irrelevant to our focus on forensic medicine education and 319 ineligible publication types. The remaining 137 full-text articles were thoroughly assessed against predefined inclusion criteria. We excluded 96 articles because they either: (1) contained incomplete datasets and (2) were conference abstracts without sufficient methodological details or full text availability. After rigorous evaluation, 41 studies were found to comply with all inclusion requirements and were subsequently incorporated into this systematic review (Figure 1).

## 3 Practical applications of artificial intelligence in forensic medicine teaching

### 3.1 Virtual anatomy and three-dimensional reconstruction

**FIGURE 2:**
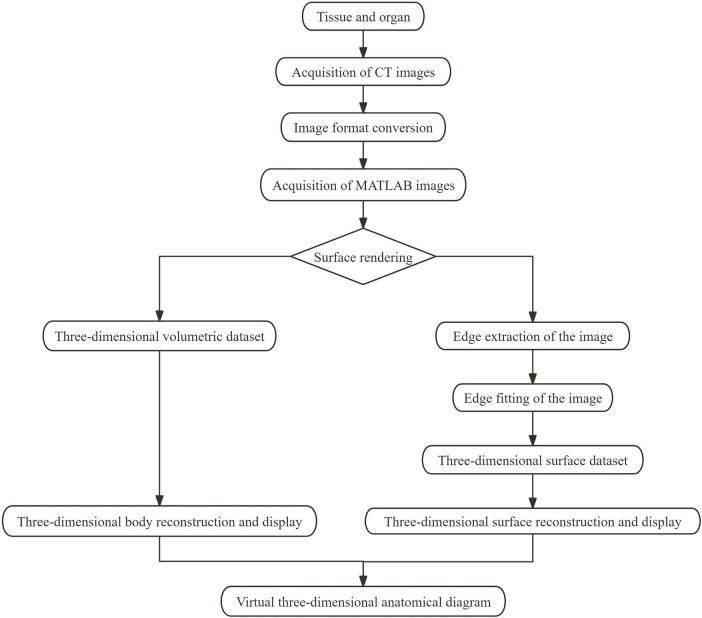
Workflow for virtual anatomical 3D reconstruction from CT imaging to model visualization.

Anatomy, as a foundational discipline in forensic medicine, has traditionally faced ethical concerns, limited access to cadaveric specimens, and inadequate simulation of pathological conditions (6). In recent years, advancements in computer vision, artificial intelligence, and medical imaging have enabled the development of novel virtual anatomy techniques. These techniques primarily involve the integration of multimodal imaging data to construct high-precision 3D anatomical models, which are further enhanced through interactive segmentation and physiological simulation capabilities (Figure 2). Building on these advancements in virtual anatomy techniques, the process begins by selecting organs relevant to forensic medicine teaching. Once selected, CT scanning is performed, generating a series of continuous, high-resolution two-dimensional cross-sectional grayscale images (7). The original image data exported from CT is converted into a universal format that MATLAB can read and process. MATLAB then stacks all the two-dimensional images in sequence and builds a 3D volume data matrix in memory. Subsequently, a continuous virtual 3D anatomical diagram composed of numerous images is directly generated through surface rendering. The image boundaries are then modified through image boundary fitting. Finally, a complete, closed, and grid-based 3D model of the anatomical organ is constructed by integrating 3D reconstruction technology. In the realm of 3D reconstruction, deep learning-based voxel modeling has emerged as the predominant approach for creating high-fidelity anatomical models. For instance, the modern VGGT model utilizes a pure feedforward transformer architecture to perform 3D geometric reasoning based on single or numerous input images (8). This research has been proven capable of quickly generating 3D imaging of the crowd at tourist attractions, which can be used for real-time monitoring of the population density at tourist sites. It also has great potential for use in forensic medicine teaching. However, no studies have yet utilized the VGGT model in forensic medicine teaching, and further research is needed in this regard. Another widely adopted method is multi-view stereo vision, which simulates visual disparites using two or more cameras and reconstructs image surfaces through feature matching algorithms (9). This algorithm not only can reconstruct a highly complete model, but also has low computational cost and good generalization ability. It can be applied to small-scale platforms with limited computing resources and is more suitable for forensic medicine teaching. However, the test data in the article is limited, so this result still needs further verification.

This potential for broader application is already being realized in contemporary practice. Virtual dissection and 3D reconstruction technologies are now being implemented in forensic practice and university education worldwide. Pringle et al. (10) explored the integration of 3D virtual imaging technology into forensic medicine education by establishing virtual anatomy laboratories, simulated outdoor environments, and interactive educational video games. The implementation of these technologies has contributed to the development of a sustainable and practical learning environment. However, the study was limited to a single class comprising 40 students, representing a relatively small sample size; thus, the findings require validation through larger-scale experimental studies. Similarly, Darras et al. (11) introduced virtual anatomy technology in anatomical instruction. Their results indicated that 78.7% of students perceived an enhanced understanding of cadaveric anatomy and its clinical applications through virtual learning. Although this study involved a larger sample of 202 participants, data collection relied solely on student self-reported surveys, without objective assessment of knowledge acquisition or skill development. Although 3D reconstruction technology is gradually being applied across many fields of forensic medicine teaching (Table 1), several significant limitations remain. First, a major issue lies in the deplorable adaptability of current systems to complicated pathological conditions. Given that forensic medicine involves a wide range of complicated cases where injuries vary significantly among victims, the virtual anatomical or pathological models generated by these technologies often fail to accurately represent complex pathological structures. Second, another limitation lies in that it is expensive to acquire high-quality medical imaging data and it is costly in terms of substantial computational resources to train 3D models. These high costs hinder their extensive adoption, particularly in resource-limited universities (12, 13). Finally, because virtual dissection lacks the tactile feedback provided by physical dissection, students may develop an incomplete appreciation for the sanctity of human life (14).

**TABLE 1:** The application of 3D reconstruction technology in forensic forensic medicine teaching.

Applied subject	AI applications	Improvement effect	References
Forensic physical evidence	3D virtual simulation of crime scenes and forensic labs	Enhanced ability to obtain physical evidence and perform evidence extraction procedures	(15)
Forensic anthropology	3D modeling of human bones at various postmortem intervals	Improved understanding of osteology and visualization of taphonomic changes	(16)
Forensic scene investigation	VR-based simulation of various crime scenarios	Enriched first-hand investigative experience and improved evidence collection skills	(17)
Forensic scene investigation	3D laser scanning for virtual outdoor crime scene reconstruction	Immersive engagement with realistic scenarios, leading to enhanced practical skills	(18)
Forensic pathology	Photogrammetry-based 3D autopsy case modeling	Direct visual understanding of postmortem findings across causes of death, supplementing traditional autopsy teaching	(19)
Forensic anthropology	3D imaging for skeletal reconstruction	No significant difference between the use of real bones for demonstration in the laboratory and 3D imaging technology in the learning effect.	(20)
Forensic physical evidence	Virtual development of DNA laboratory	Enabling students to conduct forensic evidence experiments without having to go to the laboratory	(21, 22)
Forensic pathology	3D recording and VR/animation processing of forensic neck dissections	3D visualization of regional anatomy and dynamic procedural demonstration	(23)
Forensic pathology	Integration of virtual anatomy technology into teaching	Enhanced integration of theoretical knowledge with practical application	(24)

### 3.2 Intelligent case repository and simulation teaching system

Traditional teaching process in forensic medicine education largely relies on case-based instruction. However, due to constraints such as case confidentiality, fragmented resource distribution, and limited hands-on practice, forensic medicine teaching often struggles to access real case materials, involves high operational risks, and suffers from low-fidelity scene reproduction (25, 26). With advancements in artificial intelligence and virtual reality, a dual-core teaching system combining “intelligent case repository” and “simulation teaching system” has emerged (27, 28). In the future, forensic medicine teaching can also utilize this technology to collect real case data and establish three-dimensional models. Then, these models are used to develop a comprehensive platform for forensic medicine teaching that encompasses forensic pathology, forensic evidence examination, and crime scene reconstruction.

At the core of this system is the construction of a library of forensic intelligent cases that relies on the collection and standardization of multimodal data. Information from documented past cases—such as case descriptions, autopsy images, scanned physical evidence, and on-site investigation records—should be integrated into the database and annotated using multi-label classification techniques (29). Concurrently, an interdisciplinary knowledge graph should be developed to connect knowledge from anatomy, pathology, and forensic medicine, that can enable cross-case knowledge transfer and supporting cross-case reasoning. The theory of experiential learning emphasizes learning through a cycle of specific experiences, reflective observation, abstract conceptualization, and active experimentation. However, fully implementing this cycle in traditional forensic education has been challenging due to the constraints of reality, resources, and safety. AI technology, emerging in this context, provides an unprecedented path to realize this theoretical model. The system that simulates virtual reality crime scene can generate an infinite variety of virtual scenarios. Under this system, students can immerse themselves in a highly realistic 3D environment by wearing VR equipment and conduct on-site investigation practices.

**FIGURE 3:**
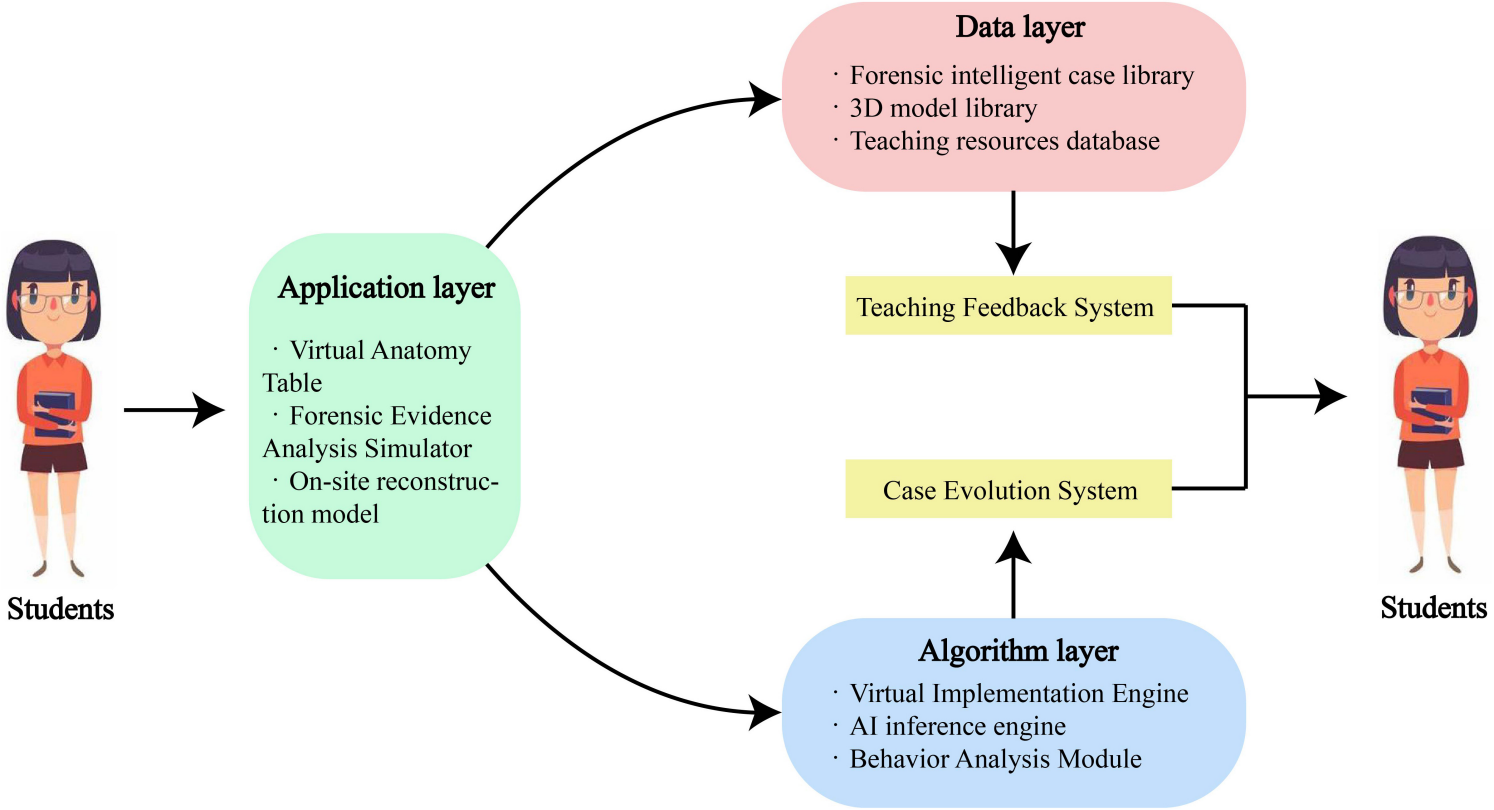
The structural framework of the simulation teaching system.

In teaching, the intelligent case library should be integrated with a simulation-based teaching system. Such a system comprises three main components, including the data layer, the algorithm layer, and the application layer (Figure 3). The database of the future forensic medicine simulation teaching system can be built upon the foundation of the forensic intelligent case database, and it includes a three-dimensional model library and a teaching resource library. The algorithm layer serves as the core technical component of the simulation-based teaching system, primarily utilizing a combination of “virtual reality engine + AI reasoning engine + behavior analysis module” to enable dynamic interpretation of case scenarios in a virtual environment. The algorithm layer can provide simulated crime scenes for forensic education and analyze the students’ operational processes during the on-site investigation. Finally, the application layer comprises tools such as a virtual autopsy table, an evidence analysis simulator, and a scene reconstruction sandbox. These tools bridge real case data with immersive simulation, allowing trainees to perform virtual autopsies using haptic feedback devices. Trainees’ procedural steps and diagnostic reasoning are then synchronized and compared with AI-generated reference solutions. This enables the automated generation of multidimensional evaluation reports to assess student learning outcomes (30, 31). Currently, several institutions have begun developing forensic simulation teaching systems. Mayne et al. (17) developed a virtual crime scene investigation laboratory using 3D imaging technology, enabling students to wear head-mounted display devices and conduct immersive investigations within a simulated crime scene. Participants reported high levels of satisfaction, and notable improvements in forensic investigation skills were observed. Nevertheless, the study included only 10 cases, limiting statistical robustness, and some students experienced adverse physical reactions such as dizziness during the experience. Darlene et al. (24) implemented virtual imaging technology to create a virtual autopsy laboratory, where students demonstrated improved proficiency in autopsy procedures, with nearly 80% achieving competent performance. This study shared similar limitations with prior research, including a small sample size and reports of motion-related discomfort among users. Collectively, these studies indicate that simulation-based teaching systems are increasingly being adopted in forensic medicine education and show promise in enhancing learning outcomes. However, due to current technological constraints, virtual reality remains insufficient to replace traditional hands-on training and should be regarded as a supplementary tool rather than a primary instructional method. Widespread adoption as a core teaching approach is still distant.

### 3.3 Personalized evaluation of teaching and learning

#### 3.3.1 Practical scenarios of AI for personalized learning

AI-driven simulation technology can convert real crime scenes or anatomical scenarios into virtual simulations, enabling learners to engage in repeated, self-directed practice in observation, operation, and study in a risk-free setting. The theory of deliberate practice-a theory that emphasizes purposeful and feedback-driven targeted training outside the comfort zone-has found powerful enabler in AI technology, allowing forensic skills training to achieve unprecedented precision (32, 33). Throughout the learning process, instructors can dynamically readjust the teaching contents and difficulty levels according to their students’ mastery of key concepts and mistakes students make during operations. More crucially, AI fulfills the core tenet of deliberate practice—immediate feedback. In the future, natural language processing technology can be integrated to provide real-time responses to students’ questions, effectively eliminating the inherent feedback delay in traditional teaching. By leveraging natural language processing, the system provides real-time responses to student inquiries, effectively dismantling the feedback delays inherent in traditional instruction and allowing for continuous skill refinement.

#### 3.3.2 Innovative practices in AI-driven teaching evaluation

Post-class teaching assessment constitutes a critical component of the educational process. Accurately evaluating students’ engagement with and comprehension of course material is essential for effective pedagogical evaluation. Traditional written examinations are inadequate for assessing the practical competencies required of forensic professionals. In contrast, AI systems offer significant potential in this domain. AI can collect and analyze interaction data generated during students’ virtual experiments, generating visualized reports on learning performance. Through comprehensive analysis across diverse learner groups, AI enables the identification of common learning difficulties, allowing educators to obtain intuitive, accurate, and timely feedback on teaching effectiveness. To date, however, no studies have attempted to integrate AI systems into post-class assessment within forensic education. Further research and development in this area are necessary to realize its full potential.

## 4 Paradigm shift: AI-driven transformation in forensic education

### 4.1 From “passive reception” to “interactive practice”

The constructivist learning theory holds that knowledge is not passively received but is actively constructed by learners based on their experiences (34). The introduction of AI also promotes this active process of knowledge construction. With the continuous AI development, the educational model in forensic medicine in the future will evolve from the traditional “teacher-student” binary structure to a “teacher-machine-student” tripartite model. This shift signifies a transition from the knowledge-based “passive reception” paradigm to a personalized “interactive practice” one that integrates teaching, learning, and practical application, powered by AI. In the future, this interactive teaching model will have the following characteristics. First, AI can construct highly realistic virtual environments, overcoming time and space constraints. The integration of virtual simulation experiments into the curriculum enables students to safely and repeatedly practice skills like fingerprint collection and ballistic trajectory determination, thereby significantly enhancing their on-site investigation capabilities (35). Second, AI personalizes learning pathways through student data analysis and dynamic feedback, enabling “data-driven” personalized instruction. During the student learning process, AI generates customized training plans based on students’ operational data. Additionally, AI can generate virtual cases of varying difficulty levels, ranging from basic poisoning identification to complex multi-cause death analysis, catering to diverse and personalized learning needs. The theory of mastery learning holds that as long as sufficient time and appropriate conditions are provided, the majority of students can master the learning content (36). The AI-driven personalized learning system provides the operational framework to realize this principle in forensic medicine education. Third, AI allows forensic medicine to move toward practical, interdisciplinary collaboration with law, computer science, and data science by facilitating real-scenario integration. Similarly, authentic legal cases can be introduced as teaching examples, with AI providing structured guidance and performance evaluation for students.

### 4.2 Shift of content from “single discipline” to “multidisciplinary integration”

The focus of forensic medicine courses has been on core domains such as forensic pathology, toxicology analysis, and evidence technology, while limited integration with fields like computer science and data science remained limited. The onset of AI has fostered interdisciplinary interaction between forensic medicine and fields like computer science and data science. This development necessitates forensic professionals not only to master forensic medicine knowledge but also to acquire expertise in computer science, biology, and law. To cultivate interdisciplinary professionals skilled in “medicine + technology + law,” modern forensic medicine teaching should incorporate foundational knowledge such as algorithm principles and data ethics. The iterative updating of AI-driven teaching content can be achieved through a dynamic closed-loop process of “technology evolution monitoring → case library update → teaching module adjustment” (37). The proposed curriculum reconfiguration will rest on a foundational framework comprising forensic medicine principles, biostatistics, and Python programming in the future. It will integrate core technical components such as forensic image processing, biometric recognition algorithms, and judicial big data analysis. The curriculum bridges classroom instruction and real-case scenarios through key integrative components such as virtual crime scenes and AI-assisted report writing. Furthermore, it is recommended to implement a “dual mentor system” involving both forensic medicine faculty and AI engineers in the teaching faculty.

### 4.3 Toward a paradigm of process-oriented assessment: from terminal exams to continuous competency profiling

**FIGURE 4:**
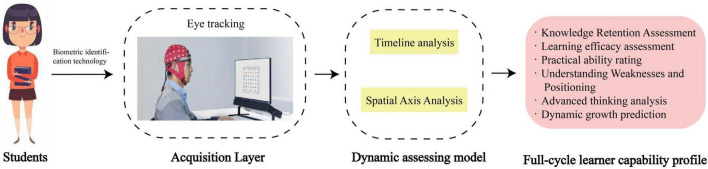
Intelligent evaluation architecture framework for multimodal data fusion.

At present, forensic medicine education assessment is primarily built on final written exams and experimental reports (38). However, this approach struggles to evaluate critical forensic competencies such as students’ capacity for critical reasoning in crime scene reconstruction and the hypothesis verification process in forensic evidence analysis. To address these limitations, it is urgently necessary to establish an intelligent assessment system based on multimodal data fusion. This system can adopt a paradigm of process-oriented assessment, namely, a shift from the traditional terminal exams to continuous competency profiling (Figure 4). This assessment requires the integration of biometric recognition, learning behavior modeling and knowledge graph tracking technologies (39). It enables a more comprehensive judgment of whether students have acquired the required forensic competencies. Its technical framework can be broadly categorized into three components, including a multimodal data collection layer, a dynamic assessment model, and the biometric recognition technology. The multimodal data collection layer can be constructed by integrating data from four sources: eye-tracking, speech semantics, experimental operations, and digital footprints. During the assessment, Biometric recognition technology is used to input the collected data into the dynamic assessment model. Then, a 2-D assessment framework based on a time-axis row and a space-axis column is established to serve as the foundation for subsequent comprehensive evaluation. Along the time-axis, the system records students’ learning trajectory, while on the space-axis, it maps the knowledge nodes they have mastered. By analyzing students’ operation trajectories, critical reasoning in decision-making, and time spent on different tasks within the virtual environment, the system provides a holistic evaluation of their progression from knowledge acquisition to skill application. Currently, this intelligent assessment system is still in the theoretical stage. Some technologies are not yet capable of meeting the requirements of this intelligent assessment system. However, in the future, it is certain to become a hot topic in forensic education research.

## 5 Navigating tensions: ethical, technical, and pedagogical considerations

### 5.1 Technical and infrastructural barriers

As AI seeks deeper integration into forensic medicine education, data barriers have emerged as the primary obstacles to leveraging AI in forensic medicine education. Due to the sophisticated and diverse nature of forensic medicine scenarios, it is difficult to collect certain forensic data such as corpse images, DNA samples, and crime scene records. Moreover, data annotation can only be performed by professional forensic experts, which increases both cost and subjectivity of the annotation process. The inherently poor interpretability (“black box” nature) of AI algorithms and the relatively low level of confidence and trust in AI also undermine the adoption of AI in forensic medicine teaching (40, 41). This field demands that conclusions be scientifically valid and legally defensible. However, current AI models often function as “black boxes,” obscuring the internal reasoning process and limiting both transparency and interpretability. This This opacity may lead to misjudgements caused by biased training data. Such misjudgements risk distorting students’ foundational understanding of forensic reasoning. Aydogan et al. (42) found that demonstrated that ChatGPT-4 produced forensic reports with high accuracy, albeit slightly lower than that of human experts, underscoring ChatGPT’s limitations in handling forensic cases. However, the cases used in the article were relatively simple. In reality, when dealing with complex forensic incidents, the accuracy of AI still needs further research. Finally, the limited infrastructure of many educational institutions, particularly in terms of hardware resources and computing power, further constrains the application of AI in forensic medicine education. Within these constrained applications, AI is primarily used to process and reconstruct high-resolution images in forensic medicine teaching environment. While current technologies can meet basic needs for image recognition, challenges remain in areas such as augmented reality systems, particularly in capturing high-quality images under poor lighting conditions.

### 5.2 Ethical, legal, and pedagogical risks

AI systems in forensic education are increasingly attracting attention with regard to liability. Article 2 of China’s “Interim Measures for the Administration of Generative Artificial Intelligence Services” states that “industry organizations, enterprises, educational and research institutions, public cultural institutions, and relevant professional bodies that develop and apply generative artificial intelligence technologies but do not provide such services to the domestic public are not subject to these measures” (43). Compared to educational AI, operational forensic AI requires even higher standards. The EU’s “Artificial Intelligence Act” explicitly classifies AI systems used in judicial and law enforcement contexts as high-risk, mandating strict compliance with requirements such as risk management systems, high-quality datasets, activity logging, comprehensive documentation, transparent user information, human oversight, and robustness, accuracy, and cyber security standards (44). While educational AI is included within the broader regulatory framework, its specific obligations remain significantly more lenient, focusing primarily on data privacy and educational equity.

Data governance and privacy constitute fundamental challenges in the development of forensic education AI. Teaching in this domain depends heavily on real-world case materials, which often involve sensitive information related to the dignity of the deceased, personal privacy, and judicial confidentiality principles. To address these concerns, a tiered and categorized data governance framework must be established. First, clear legal norms should govern the custody and use of forensic data. Second, technical solutions such as data de-identification and synthetic data generation should be advanced to build simulation databases that protect privacy while preserving educational value. Additionally, formal consent mechanisms and flexible frameworks for the ethical use of human remains and case records in education must be developed to ensure both legal compliance and procedural transparency.

Algorithmic bias and limited explainability represent core risks in forensic education AI. Bias in algorithms may result in inaccurate assessments during training, while poor model interpretability undermines confidence in AI-assisted analytical conclusions. A dual strategy is required to mitigate these risks. First, institutions should strengthen the detection of algorithmic bias and assess its impact on educational outcomes by integrating algorithm auditing and fairness evaluation into curricula, enabling learners to understand how biases emerge. Second, efforts must focus on enhancing the explainability of AI models within judicial contexts. Through targeted instruction, students should develop the ability to critically evaluate the reasoning behind AI-generated outputs, assessing their reproducibility and interpretability according to evidentiary standards.

Teaching dependence presents a practical challenge in forensic education. Forensic medicine relies extensively on authentic case data for hands-on instruction. This reliance, coupled with restricted access to real data due to privacy and legal constraints, not only limits pedagogical effectiveness but also prevents students from experiencing realistic scenarios involving AI-assisted forensic practice. Only through access to sufficient, compliant simulated case data can AI technologies be effectively integrated into forensic education, thereby avoiding theoretical detachment from actual practice.

The erosion of professional judgment poses a potential crisis in the integration of AI into forensic education. Overreliance on opaque AI outputs during model development and deployment may lead to two critical issues: first, undetected errors may become entrenched and perpetuated, compromising analytical accuracy; second, practitioners’ capacity for independent professional judgment may deteriorate, fostering uncritical dependence on technology. To prevent this, forensic AI education should promote interdisciplinary curriculum design and ethical standardization, jointly establishing ethical guidelines and operational protocols for AI use. Furthermore, through case-based teaching and structured ethical discussions, students’ abilities in holistic judgment and professional accountability should be strengthened, ensuring that technological tools support—rather than supplant—professional expertise.

### 5.3 Shortage of interdisciplinary AI talents in forensic medicine education

Since integration of AI into forensic medicine education necessitates collaboration across various disciplines, including medicine, computer science, and law, educators involved in forensic medicine must possess knowledge in these areas. However, this is compounded by a mutual knowledge gap: most forensic educators lack AI expertise, and computer scientists seldom grasp the nuances of forensic medicine. Johnson et al. (45) conducted a questionnaire survey among 161 staff members and graduate students engaged in forensic dentistry. The feedback indicated that dental professionals and graduate students had limited knowledge of AI technologies such as VR and 3D. This disconnect impedes the alignment of technology with pedagogical needs.

While exploratory projects in AI-assisted forensic medicine education have emerged at some institutions, the broader field still urgently requires the systematic development and expansion of interdisciplinary curricula across universities to cultivate professionals with the necessary interdisciplinary expertise.

## 6 Discussion

With the continuous advancement of AI technology and its growing integration with related disciplinary domains, the shift of forensic medicine education toward intelligent, immersive, and lifelong learning paradigms has become an inevitable trend. The incorporation of AI not only reconfigures teaching scenarios and modes of knowledge delivery but also extends into judicial practice and social governance, thereby continuously expanding the functional scope and societal values of forensic medicine education. For case generation and intelligent feedback, AI demonstrates strong capabilities in integrating and analyzing multi-source data for forensic instructional purposes. Leveraging this capacity, future forensic instruction can transcend the traditional disciplinary boundaries defined by anatomy and pathology. Specifically, it can construct highly realistic virtual crime scenes by fusing multimodal data—such as images, biological signals, and environmental information-thereby significantly enriching the diversity and realism of curricular offerings and practical training. AI is particularly well-suited to the flipped classroom approach, a pedagogical model where its capabilities can be fully leveraged. Unlike the conventional flipped classroom model reliant on passive video-based autonomous learning, the AI-enhanced version enables highly tailored learning trajectories for each student—a “one person, one path” model. By employing 3D modeling and AI-driven analytical software, forensic medicine education can design customized training modules tailored to individual learning needs, while adaptive algorithms identify knowledge gaps and deliver targeted materials to address them effectively.

Alongside professional training, AI technologies have significantly promoted the dissemination of forensic knowledge to the public. This is made possible by technologies such as generative AI and extended reality, which can be leveraged to develop accessible pedagogical tools for lay audiences. For example, legal education chatbots based on systems like “Xiaofa Shuidi” can walk lay audiences and users through forensic cases in clear and lay-friendly language. Similarly, VR modules can immerse the public directly in crime scene investigations. This first-hand exposure not only demystifies the expert’s role but also corrects common misconceptions perpetuated by films. These tools also hold potential for integration into primary and secondary STEM curricula and for fostering early interest in forensic science careers among youth.

Nevertheless, several limitations remain in current AI applications to forensic medicine education. First, some AI-enabled imaging technologies carry inherent health risks—for instance—for example, dizziness or discomfort from VR headsets—which can directly impair learning outcomes. In a sense, ensuring broad accessibility and tolerance of 3D imaging–based instruction remains a critical challenge for future research and use of VR headsets in forensic medicine education. Second, given that AI technologies are not yet fully mature, complex tasks such as 3D anatomical reconstruction and experimental procedure analysis may not be accurate enough and thus may lead to student misinterpretations and affect their practical skill development. Therefore, in AI-integrated teaching environments, it is essential to establish expert review mechanisms to ensure scientific validity alongside efficiency gains. Third, the “black box” nature of many AI models limits their transparency, making it difficult for learners to understand the rationale behind model predictions. This opacity hinders students’ ability to grasp the model’s decision-making logic. To address this, future research should focus on developing interpretable AI models—such as those incorporating heat map annotations—that generate explanations for their decisions, thereby enhancing pedagogical transparency and conceptual understanding.

## 7 Conclusion

Artificial intelligence has introduced unprecedented opportunities for forensic medicine teaching, ushering it into an era of greater intelligence, personalization, and efficiency. Its values extend beyond the innovation of technical tools to the shift of educational paradigms—from “knowledge transmission” to “ability cultivation.” However, technological empowerment must align with the core principles of forensic science to avoid the trap of “AI for the sake of AI” and the related pitfall of “garbage in, garbage out,” where biased or poor-quality data undermine the system’s scientific and ethical foundation. Future studies must strike a balance between technological innovation, ethical standards, and educational equity, building a next-generation forensic medicine education system that integrates human-machine collaboration and seamlessly combines virtual reality and real-world experiences.

## Footnotes

1
https://pubmed.ncbi.nlm.nih.gov/

2
https://www.cnki.net/

3
https://scholar.google.com/

4
https://www.sciencedirect.com/

5
https://onlinelibrary.wiley.com/

6
https://mjl.clarivate.com
